# Association Between Iron Deficiency Anemia and Dental Caries in Children: A Systematic Umbrella Meta‐Analysis

**DOI:** 10.1111/jhn.70149

**Published:** 2025-10-27

**Authors:** Mohammed Taib Fatih, Mohammed Khalid Mahmood, Yad Mariwan Mohammed Ali, Tara Ali Rasheed, Zana Fuad Noori, Handren Ameer Kurda, Mohammed Aso Abdulghafor, Balen Hamid Qadir, Hevi Nihad Mohammed Fadhil, Herve Tassery, Delphine Tardivo, Romain Lan

**Affiliations:** ^1^ Department of Dentistry Komar University of Science and Technology Sulaymaniyah Iraq; ^2^ Faculty of Medical and Paramedical Sciences, Aix‐Marseille University, French National Center of Scientific Research (CNRS), French Blood Establishment (EFS), Bio‐Cultural Anthropology, Law Ethics and Health Laboratory (ADES) Marseille France; ^3^ Department of Dentistry Tishk International University Sulaymaniyah Iraq; ^4^ College of Dentistry Sulaymaniyah University Sulaymaniyah Iraq; ^5^ College of Dentistry American University of Sulaymaniyah Iraq AUIS Sulaymaniyah Iraq; ^6^ Dental School of Medicine, Conservative and Endodontic Department Aix‐Marseille University Marseille France; ^7^ Marseille Hospital APHM IHU‐MEPHI Institute Marseille France

**Keywords:** anemia, dental caries, early childhood caries, haemoglobin, iron deficiency anemia, meta‐analysis, systematic review, umbrella review

## Abstract

**Background:**

Dental caries and iron deficiency anemia (IDA) are prevalent especially in developing children. There seems to be an association between these two variables.

**Objective:**

To evaluate this association among children and adolescents using a systematic review and umbrella meta‐analysis technique.

**Methods:**

The PRISMA reference databases like PubMed, Scopus, Web of Science and Google Scholar were searched for all records published before January 2025. Meta‐analyses that evaluated the association among < 18‐year‐olds were included. Parameters like odds ratio (OR) and mean difference (MD) with the 95% confidence interval (CI) were used to compare between the groups. *I*² and Cochran's Q (*χ*²) tests were used for measuring heterogeneity, whereas Egger's test was utilised to measure publication bias. A Measurement Tool to Assess systematic Reviews (AMSTAR 2) was used to assess the quality of the meta‐analyses. The degree of certainty of the outcomes was evaluated using the Grading of Recommendations Assessment, Development and Evaluation (GRADE) tool.

**Results:**

A total of five meta‐analyses were included. Children with IDA were significantly over three times more likely to have dental caries than children without anemia (OR = 3.64, 95% CI: 2.45 to 5.40, *p* < 0.0001. Heterogeneity: *I*² = 80%, *p* = 0.007. Publication bias: *p* = 0.14. GRADE: Moderate). The pooled analysis for serum ferritin showed a tendency toward lower ferritin level in children with caries; but without statistical significance (MD = −3.96, 95% CI: −8.48 to 0.57, *p* = 0.087. Heterogeneity: *I*² = 66.3%, *p* = 0.031. Publication bias: *p* = 0.003. GRADE: Very low). Children with dental caries had lower haemoglobin levels, but the finding was not significant with high level of heterogeneity and publication bias (MD = −2.20 g/dL, 95% CI: −4.59 to 0.19, *p* = 0.071. Heterogeneity: 80.%, *p* = 0.006. Publication bias: *p* = 0.005. GRADE: Very low). Children with caries had lower MCV, but this result was non‐significant with evidence of heterogeneity and publication bias (MD = −1.96 fL, 95% CI: −4.02 to 0.10, *p* = 0.062. Heterogeneity: *I*² = 57.2%, *p* = 0.096. Publication bias: *p* = 0.041. GRADE: Very low).

**Conclusion:**

Dental caries was more prevalent among anemic children compared to non‐anemic ones with a low level of certainty.

## Summary

- Children with iron deficiency anemia were over three times more likely to have dental caries than children without anemia (OR = 3.64).
- Individual hematological parameters like haemoglobin, ferritin, and MCV showed non‐significant trends toward being lower in children with caries.
- The study proposes that iron deficiency and dental caries influence each other through impaired salivary function, reduced iron's antibacterial properties, dietary changes from tooth pain, and chronic inflammation.

## 1 Introduction

Dental caries is a bacterial and infectious disease characterised by progressive destruction of the tooth structure. Cariogenic bacteria in dental biofilms consume carbohydrates as an energy source. One of the byproducts of this reaction is acid [1, 2]. Especially in the absence of regular oral and dental hygiene, this acid dissolves the tooth architecture. Once the enamel, the outer layer of crowns of teeth is demineralised and decalcified, it cannot regenerate, since it lacks blood supply and nerves. Excessive caries, bacteria and bacterial products may reach the deeper layers like dentin and pulp, peri‐apical area, periodontium and even muscles and bones of the jaw [3, 4].

Despite a decrease in the prevalence and severity of dental caries worldwide in the last decades [5, 6, 7], still it affects millions of people, causing a wide spectrum of outcomes like pain, tooth loss, deterioration in the facial esthetics, initiation and/or aggravation of some systemic infections, reduction in quality of life, decrease in self‐esteem, loss of income and productivity [8, 9].

Iron is integral to numerous metabolic processes, including DNA synthesis, electron transport, and oxygen transport, rendering it essential for all living organisms [10]. Iron is necessary for the human body to manufacture myoglobin, haemoglobin of the red blood cells, and some other important hormones. Iron deficiency anemia (IDA), which is still the most prevalent nutritional deficiency globally, is the most serious effect of iron insufficiency [11]. IDA has a complex etiology, but it usually arises when the body's iron requirements are not satisfied by iron absorption, for whatever reason. Inadequate intake, decreased transport or absorption, physiological losses related to chronological, or chronic blood loss as a result of diseases are among the common causes of IDA [12, 13].

Both dental caries and IDA are prevalent especially in developing children. ECC continues to be the most common disease in children, affecting over 600 million children globally and having a major social impact [14]. According to the World Health Organization (WHO), iron insufficiency is the most prevalent and pervasive nutritional deficit, affecting 1.62 billion people worldwide, with preschool‐aged children having the highest prevalence [15]. A bidirectional association between dental caries and IDA can be proposed in which severe dental caries may cause malnutrition and iron deficiency by impairing chewing ability and nutrition intake in one hand; on the other hand, malnutrition and IDA may initiate or worsen dental caries [16]. Furthermore, it has also been suggested that iron may prevent tooth decay by inhibiting cariogenic microorganisms [17].

Serum iron level and its hematological parameters have been studied in relation to dental caries in different populations and study designs. Primary research results (mostly cross‐sectional and case‐control studies) indicated that children with a sufficient serum level of iron have a better dentition with lower chances of dental caries [18, 19, 20, 21]. Recently, several meta‐analyses were conducted in this field that helped in a better understanding of this association [15, 16, 22, 23, 24].

The aim of this study is to provide up‐to‐date evidence on the association between IDA and dental caries to dentists, clinicians and researchers through a systematic umbrella review which takes places at the top of evidence hierarchy synthesis. The results of this article could assist healthcare professionals and policymakers in identifying individuals at risk and implementing interventions to optimise outcomes in the battle against anemia and dental caries.

## 2 Materials and Methods

### 2.1 Protocol and Registration

This umbrella review was performed in accordance with the 2020 Preferred Reporting Items for Systematic Reviews and Meta‐Analyses (PRISMA) guidelines [25], and relevant methodological recommendations for umbrella reviews [26, 27]. The protocol for this review was prospectively registered in the International Prospective Register of Systematic Reviews (PROSPERO) with the registration number CRD420251007570.

### 2.2 Eligibility Criteria

This umbrella review included meta‐analyses and systematic studies that examined the relationship between childhood dental caries and IDA. The only reviews that qualified were those based on observational studies with cross‐sectional, case‐control, or cohort study designs. There were no restrictions on the observational study design within the included reviews.

Qualifying studies included children (aged 0–18 years) of any sex, geographic area, or medical care setting. IDA must have been diagnosed using clinical or laboratory diagnostic criteria from the original studies such as haemoglobin, serum ferritin, serum iron, and mean corpuscular volume (MCV). Reviews were included if they examined the association of dental caries in children with IDA, or if they reported mean differences (MDs) in hematological indices (e.g., haemoglobin, ferritin, MCV) between children with dental caries compared to children without dental caries.

Only systematic reviews and meta‐analyses published in peer‐reviewed journals in English language were included. Reviews that did not apply systematic methods (narrative review, scoping review, etc.), those exclusively related to adult participants, or those investigating iron status or dietary iron supplements without an outcome of dental caries were excluded. Conference abstracts, dissertations, editorials or any other evidence not published in a peer‐reviewed journal were excluded to ensure the credibility of the evidence identified.

### 2.3 Search Strategy

To find relevant systematic reviews and meta‐analyses evaluating the relationship between IDA and dental caries in children, a thorough search of the literature was undertaken. The electronic databases of PubMed, Scopus, and Web of Science were used to conduct the search, which included all records released before January 2025. Google Scholar was used to collect relevant grey literature and other related sources. A hand search of the included articles and associated reviews was conducted, and the reference lists of those articles were reviewed to capture as much of the relevant literature as possible.

A search strategy was established based on a combination of controlled vocabulary (e.g., MeSH terms) and keywords related to IDA, dental caries, and review/meta‐analysis methods. The search was adapted for each used database. The full list of search terms and combinations is presented in Supporting Information S1: Table S1. There were no restrictions on publication dates; however, only English articles that met the inclusion criteria were included.

### 2.4 Study Selection

All the records obtained from database and manual searching were uploaded into reference management software, and duplicates were excluded. Study selection was conducted in two parts by two independent reviewers (M. M. and B. Q.). The two‐part study selection process began (Part 1) with screening of titles and abstracts for potentially relevant studies based on eligibility criteria, and then (Part 2) the full‐text articles of potentially eligible studies were screened in detail for eligibility. Systematic reviews and meta‐analyses that met all eligibility criteria were selected for inclusion in the umbrella review. Disagreements during the screening process were resolved by discussion between the two reviewers. Where agreement was not reached, a third reviewer (M. A.) made the final decision.

### 2.5 Data Extraction

Data extraction was undertaken with a structured Microsoft Excel spreadsheet created for this umbrella review. Data was extracted independently by two reviewers (M. M. and B. Q.) to reduce the potential for errors and minimise variability. Discrepant data extraction was resolved through discussion, with a third reviewer (M. A.) consulted when consensus could not be reached.

To provide a structured collection of data by clinical variables/parameters, different worksheets within the Excel file were developed for IDA, serum ferritin, haemoglobin, and MCV as clinical variables. This aided in organising similar outcomes but also helped with the synthesis of findings related to hematological markers.

Study characteristics such as number of primary studies included for both qualitative and quantitative analysis, type of study design of the primary studies, sample size, population characteristics, and main statistical findings of the meta‐analyses were extracted.

The effect sizes for each outcome specifically for IDA were coded as odds ratios (OR) and 95% confidence intervals (CI), and for hematological markers (ferritin, haemoglobin, and MCV) as MDs with 95% CI, were coded from the meta‐analytic forest plots.

### 2.6 Quality Assessment

The AMSTAR 2 (A Measurement Tool to Assess systematic Reviews) tool was used to assess the methodological quality of the included meta‐analyses and systematic reviews [28]. This is a specialised tool for evaluating the methodological quality of systematic reviews that examine studies of healthcare interventions that are either randomised or non‐randomised. The 16 items in the AMSTAR 2 tool evaluate the following domains: protocol registration, thorough literature search, evaluation of the risk of bias in individual studies, and appropriate meta‐analysis techniques. The AMSTAR 2 tool assigns a quality rating of high, moderate, poor, or critically low to each review. Two reviewers (M. M. and B. Q.) carried out each evaluation separately, and any disputes were settled by discussion or, if required, involvement from a third reviewer (M. A.).

### 2.7 Data Synthesis and Analysis

To ensure methodological consistency across included outcomes, we utilised the DerSimonian and Laird random‐effects model [29]. For dichotomous outcomes, such as the association between IDA and dental caries, ORs with 95% CIs were recalculated. For continuous outcomes, including serum ferritin, haemoglobin, and MCV levels, MDs, and corresponding 95% CIs were estimated.

Heterogeneity among studies was assessed using the *I*² statistic test. A *p*‐value of less than 0.10 was considered statistically significant for heterogeneity. The *I*² values were interpreted following the guidelines from the Cochrane Handbook for Systematic Reviews of Interventions: values between 0% and 40% were considered possibly unimportant; 30%–60% as indicative of moderate heterogeneity; 50%–90% as substantial; and 75%–100% as considerable heterogeneity [30].

In addition, the presence of publication bias was assessed through statistical methods. Egger's regression test was applied to detect small‐study effects, with a *p*‐value of less than 0.05 indicating potential bias [31]. These methods provided a structured approach to assessing the robustness and reliability of the pooled estimates. All statistical analyses in this umbrella review were conducted using Cochrane's RevMan accessed online [32].

### 2.8 Certainty of Evidence Assessment

The GRADE (Grading of Recommendations Assessment, Development and Evaluation) approach was used to assess the certainty of the evidence for each of the outcomes included in this umbrella review. GRADE allows for a systematic and transparent approach to assessing the quality of evidence across studies, including factors related to study design, methodological rigor, consistency of findings, directness of evidence, and imprecision (and risk of publication bias) [33].

Despite the data included in this review coming from meta‐analyses of observational studies, which the GRADE approach would typically categorise as “low” certainty, we also applied GRADE based on modifying factors that upgrade or downgrade the certainty in the evidence. For example, we down‐graded evidence if there were outcomes with marked inconsistency in findings, serious risk of bias in the primary studies, and when effect estimates were imprecise (e.g., with large CIs). Conversely, we considered upgrading the evidence if the effect was large, there was a dose‐response relationship, or we felt all credible residual confounding would produce a lesser effect.

## 3 Results

### 3.1 Study Selection

**Figure 1:**
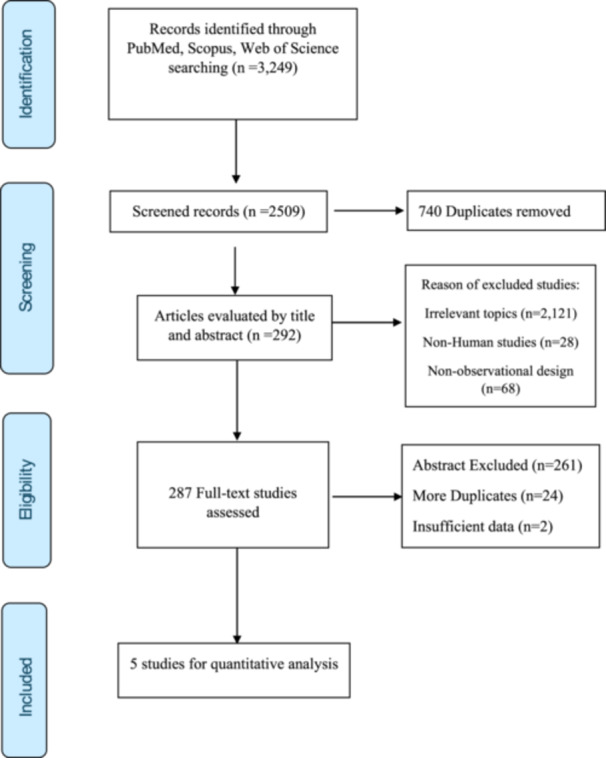
The selection process of the studies.

Through a systematic search of PubMed, Scopus and Web of Science, a total of 3249 records were detected. After deleting 740 duplicates, 2509 records remained for screening. Based on our screening of titles and abstracts, we found that 2217 articles did not meet the inclusion criteria due to reasons such as irrelevant topic (*n* = 2121), non‐human subject(s) (*n* = 28), or non‐observational design (*n* = 68). We identified 292 articles for full‐text screening and excluded an additional 286 articles because the abstract did not provide data relevant to the defined scope of the study (*n* = 261), were duplicates (*n* = 24), or had insufficient data (*n* = 2). Ultimately, five systematic reviews and meta‐analyses [15, 16, 22, 23, 24] met the inclusion criteria and were included in the final quantitative synthesis. The study selection process is shown Figure 1.

### 3.2 Characteristics of the Included Systematic Review and Meta Analysis

This umbrella review examined the relationship between IDA and dental caries, mainly in children and adolescents, including five systematic reviews and meta‐analyses. While study design and analytic methods varied, each systematic review followed a systematic approach to examine the literature, establishing eligibility criteria, diagnostic criteria for the respective conditions, and data synthesis for findings. Most of the included reviews focused on children less than 6 years of age, even though some incorporated adolescents up to 18 years of age. All the included meta‐analyses contained observational studies only, either cross‐sectional or case‐control. A total of 19,399 individuals were covered in the included meta‐analyses, of which 6803 were cases and 12,596 were controls. Table 1 presents the key characteristics of these studies.

**Table 1:** Characteristics of the included systematic reviews and meta‐analyses.

Authors (year), [Ref.]	Number of included primary studies in SR (in MA)	Study design of the primary studies	*N* (cases/controls)	Age (years)	Key findings
Aguirre‐Ipenza (2024) [22]	12 (9)	Cross‐sectional, case‐control	5957 (897/5060)	0.5–18	IDA: OR: 3.54 (2.54, 4.94) IDA: MD: 1.96 (1.07, 2.85) Hb: OR: 2.74 (1.62, 4.61)
Amrollahi (2022) [24]	9 (8)	Case‐control	1116 (689/427)	0.5–18	SF: MD: −0.23 (0.44, −0.015) Hb: MD: −0.99 (−1.81, −0.16) MCV: MD: −0.80 (−1.33, −0.27)
Easwaran (2021) [15]	14 (7)	Cross‐sectional, case‐control and cohort studies	1770 (1364/406)	0.5–6	IDA: OR: 6.07 (3.61, 10.21) Hb: MD: −0.95 (−2.53, 0.63) SF: MD: −5.31 (−12.60, 1.99) MCV: MD: −2.82 (−6.65, 1.00)
Ji (2021) [23]	12 (12)	Case‐controls	9807 (3411/6390)	0.5–7	ID: OR: 2.63 (1.85, 3.73) IDA: OR: 2.74 (2.41, 3.11) SF: MD: −5.80 (−11.97, 0.37) Hb: MD: −9.96 (−15.45, −4.46) MCV: MD: −3.72 (−6.65, −0.79)
Sharifi (2021)^a^ [16]	5 (5)	Case‐controls	749 (442/307)	2–18	SF: MD: −8.95 (−17.61, −0.29)

Abbreviations: Hb, haemoglobin; ID, iron deficiency; IDA, iron deficiency anemia; MA, meta‐analysis; MCV, mean corpuscular volume; SF, serum ferritin; SR, systematic review.

^a^ Here, we report data on serum ferritin only not the salivary iron and ferritin.

### 3.3 Characteristics of the Included Primary Studies

The included meta‐analyses of this review included 48 primary studies in their quantitative analysis on the relationship between IDA and dental caries. However, many overlapping studies were identified and only 25 unique primary articles have been covered in the included meta‐analyses.

The studies were diverse according to geographic location, study methodology, age range, sample size, and method of diagnosis for caries and anemia. The included primary papers covered approximately 20,000 individuals. Sample size varied from a minimum of 60 subjects, to a maximum of over 5000 participants from a large scale study from China [34]. The age range for study participants was largely between ages 2 and 6 years, as studies targeted preschool children, with the exception of one study that included older schoolchildren (mean age 8.87 years) [19]. The diagnosis of teeth with caries was often done using a direct clinical examination, using a standardised index to measure decay, such as Decayed, Missing, Filled Teeth (DMFT/dmft). Some studies also used an index classification system, such as Wayne's criteria for ECC [35], or S‐ECC definitions [20, 21]. Iron deficiency and anemia were mostly determined by various hematology parameters, including haemoglobin (Hb), MCV, and sometimes serum ferritin. Many studies cited WHO criteria thresholds for anemia designation, however only some studies listed laboratory reference ranges [21, 36]. Additionally, for example, one study examined ferritin concentrations with electrochemiluminescence immunoassay [35], while another study used theHemoCue system [19].

### 3.4 Quality of Systematic Reviews and Meta‐Analyses

The AMSTAR 2 instrument was utilised to evaluate the methodological quality of the systematic reviews and meta‐analyses included. All five meta‐analyses were rated as “critically low” overall quality. This indicates numerous critical weaknesses and non‐critical weaknesses across key domains. A common issue among all reviews was the lack of a study exclusion list and justification (Q7) and the failure to report funding sources in included studies (Q10). Most reviews also did not provide an adequate assessment of publication bias (Q15) even if they used appropriate methods for the statistical synthesis. Supporting Information S1: Table S2 provides a full itemised AMSTAR 2 rating for each included meta‐analysis.

### 3.5 Association Between IDA and Dental Caries

**Figure 2:**
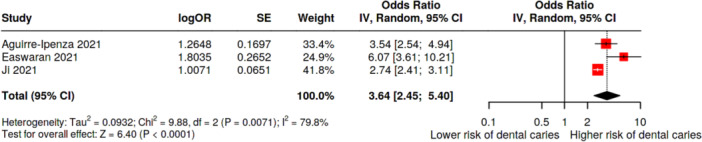
Forest plot of the association between iron deficiency anemia and dental caries. CI, confidence interval; SE, standard error.

**Figure 3:**
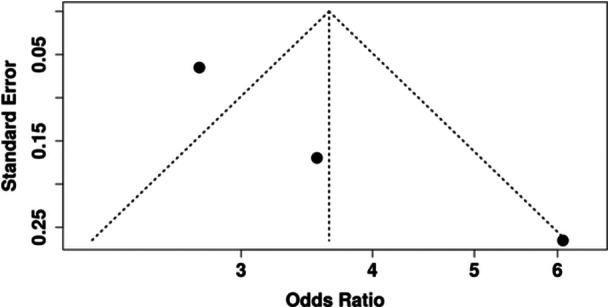
Funnel plot of the association between iron deficiency and dental caries showing no publication bias (*p* = 0.14).

A total of three meta‐analyses were included that examined the relationship between IDA and the risk of dental caries among children. The pooled analysis demonstrated a statistically significant association and children with IDA were over three times more likely to have dental caries than children without anemia (OR = 3.64, 95% CI: 2.45–5.40, *p* < 0.0001, Table 2 and Figure 2). There was considerable and statistically significant heterogeneity across the meta‐analyses (*I*² = 80%, *p* = 0.07). However, no evidence of publication bias was present (*p* = 0.41) (Table 2 and Figure 3). Based on the GRADE framework, the certainty of the evidence for this association was rated as moderate (Supporting Information S1: Table S3).

**Table 2:** Association between iron deficiency anemia, serum ferritin, haemoglobin and MCV levels with dental caries.

Exposure	Number of meta‐analyses	Effect size (95% CI)	*p*‐value	*I* ^2^, *p* heterogenicity	*p* Egger' test (publication bias)	GRADE
IDA	3	3.64 (2.45, 5.40)^a^	< 0.0001	79.8%, 0.007	0.14	Moderate
Serum ferritin	4	−3.96 (−8.48, 0.57)^b^	0.087	66.3%, 0.031	0.003	Very low
Haemoglobin	3	−2.20 (−4.59, 0.19)^b^	0.071	80.2%, 0.006	0.005	Very low
MCV	3	−1.96 (−4.02, 0.10)^b^	0.062	57.2%, 0.096	0.041	Very low

Abbreviations: CI, confidence interval; GRADE, Grading of Recommendations Assessment, Development, and Evaluation; IDA, iron deficiency anemia; MCV, mean corpuscular volume.

^a^ Odds ratio (95% confidence interval).

^b^ Mean difference (95% confidence interval).

### 3.6 Hematological Differences in Children With and Without Dental Caries

#### 3.6.1 Serum Ferritin

**Figure 4:**
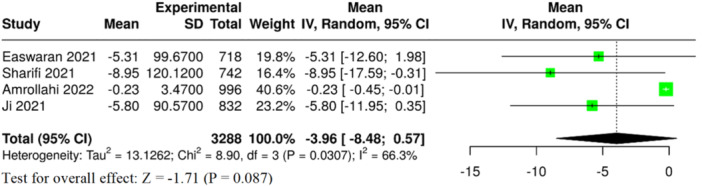
Forest plot of serum ferritin in children with and without dental caries. CI, confidence interval; SE, standard deviation.

Four meta‐analyses examined the levels of serum ferritin in children with and without dental caries. The pooled analysis presented a tendency toward lower ferritin level in children with caries; however, this finding was not statistically significant overall (MD = −3.96, 95% CI: −8.48 to 0.57, *p* = 0.087, Table 2 and Figure 4). As an important limitation, studies were found to have substantial heterogeneity (*I*² = 66.2%, *p* = 0.031), and there was evidence of publication bias (*p* = 0.003) (Table 2). Based on these limitations, the certainty of evidence according to GRADE was rated as very low for this outcome (Supporting Information S1: Table S3).

#### 3.6.2 Haemoglobin

**Figure 5:**
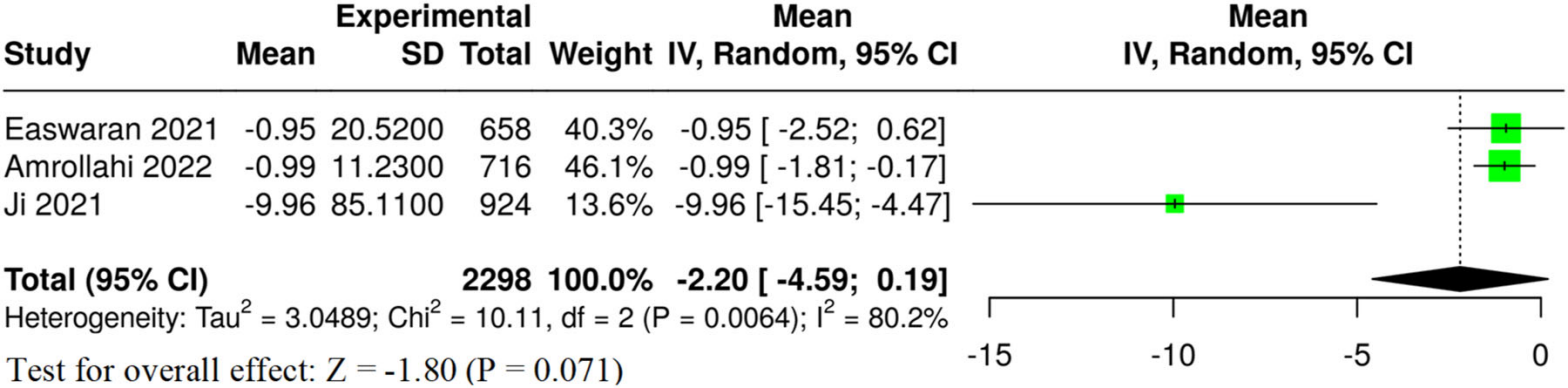
Forest plot of haemoglobin in children with and without dental caries. CI, confidence interval; SE, standard deviation.

Three meta‐analyses assessed haemoglobin levels. The pooled analysis presented a tendency toward lower haemoglobin levels in children with caries; however, this finding was not statistically significant overall (MD = −2.20 g/dL, 95% CI: −4.59 to 0.19, *p* = 0.071, Table 2 and Figure 5). The results showed high heterogeneity (*I*² = 80.2%, *p* = 0.006) and strong evidence of publication bias (*p* = 0.005) (Table 2). The overall GRADE certainty of evidence for this outcome was rated as very low (Supporting Information S1: Table S3).

#### 3.6.3 Mean Corpuscular Volume

**Figure 6:**
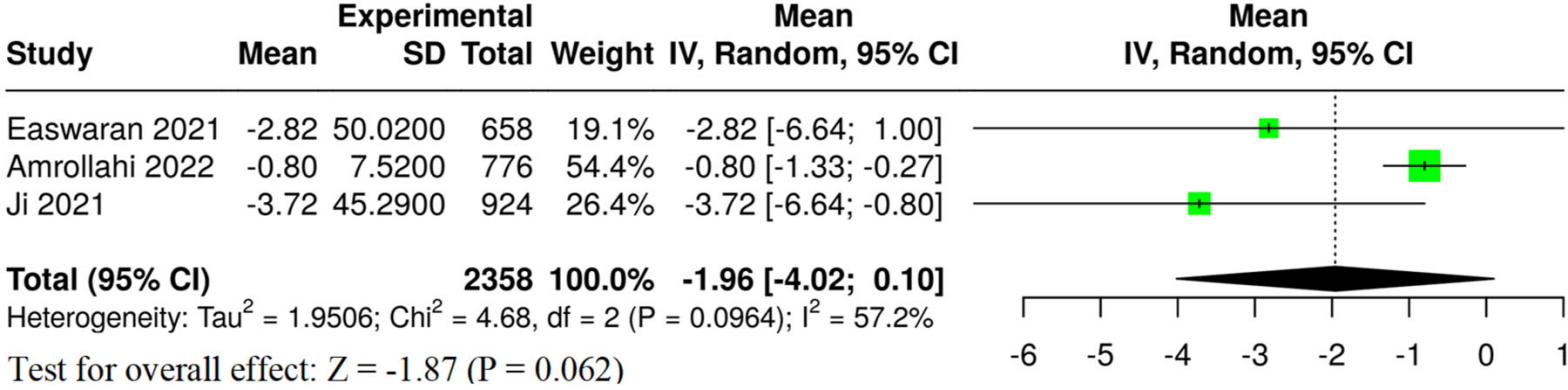
Forest plot of mean corpuscular volume (MCV) in children with and without dental caries. CI, confidence interval; SE, standard deviation.

In three meta‐analyses, differences in MCV were compared between children with dental caries and children without caries. The pooled analysis presented a tendency toward lower MCV levels in children with caries; however, this finding was not statistically significant overall (MD = −1.96 fL, 95% CI: −4.02 to 0.10, *p* = 0.062, Table 2 and Figure 6). The degree of heterogeneity was moderate (*I*² = 57.2%, *p* = 0.096) and some evidence of publication bias was found (*p* = 0.041) (Table 2). The overall certainty of evidence for this outcome was rated as very low (Supporting Information S1: Table S3).

## 4 Discussion

Dental caries is a multifactorial disease with several modifiable and non‐modifiable risk factors. The risk factors can be roughly classified into genetic and environmental. The pathogenesis of dental caries includes four major components: (1) *Host*: Dental caries manifest only in the presence of teeth. In addition, some characteristics of the dentition, like the arrangement of the teeth (presence or absence of malocclusion) may play a role. Regular dental hygiene measurements affect dental caries as an outcome. Moreover, saliva has a protective function against dental caries. (2) *Carbohydrate/nutrition*: a substrate is needed by the bacteria to survive in dental biofilms. Besides, the quality and quantity of nutrition affect this substrate. (3) *Cariogenic bacteria*: Research has shown that the consumed sugar did not lead to dental caries in germ‐free rats. (4) *Time*: The duration of the interaction of the mentioned factors determines the stage and severity of the disease [1, 3, 37, 38, 39, 40, 41, 42, 43].

Nutrition is a crucial component in growth, development, immunity and healing. Nutrition plays an important role both in health and disease states. On the other hand, Malnutrition is characterised by imbalances of vital nutrients, deficits or excesses in food intake, or inadequate nutrient use [43, 44]. Malnutrition can be measured through special tools in specific age groups. WHO roughly classifies malnutrition into two types: (1) macro‐nutrient malnutrition which includes age‐specific, weight‐ and height‐related anthropometric measurements. (2) micro‐nutrient malnutrition which is related to the deficiency or excess of important vitamins and minerals [45, 46]. The most important serum micro‐nutrients include iron, folate, calcium, zinc, potassium, albumin, vitamins A, B, C and D [47, 48, 49, 50, 51, 52].

Dental caries and IDA are prevalent especially in developing children. Both have serious impacts on affected individuals and populations. As two multifactorial parameters, IDA and ECC have a reciprocal relationship. Diet is strongly linked to both IDA and ECC independently [3, 53, 54], and there may be a connection between the two variables [24].

There was variability across primary studies in relation to IDA prevalence and the association of dental caries with IDA. For instance, Ji et al. [55] found an ECC prevalence of 80.6% among children with IDA, compared to 58.1% for the same cohort without IDA. Likewise, in the Singh et al. [18] study, 43% of the S‐ECC children had IDA, compared to only 7% in the control group. On the contrary, Shamsaddin et al. [56] indicated no substantial association between ECC and the blood indices.

To the best of our knowledge, the present study is the first systematic umbrella review on the association between dental caries and IDA. From a population and study design perspective, without exception, the included meta‐analyses of this umbrella review consisted of primarily cross‐sectional and case‐control studies, highlighting the predominance of non‐interventional studies in this area. Aguirre‐Ipenza et al. [22], included 12 studies (9 included in the meta‐analysis), which again consisted of primarily cross‐sectional and case‐control studies. Amrollahi et al. [24], examined eight case‐control studies. Easwaran et al. [15] synthesised 14 studies in their qualitative synthesis (seven included in the meta‐analysis), which consisted of cross‐sectional, case‐control, and cohort studies. Ji et al. [23] included 12 case‐control studies, while Sharifi et al. [16] used 13 observational studies, in which only 5 were related to serum ferritin.

The GRADE assessment of the included meta‐analyses concluded with low certainty of evidence for a relationship between IDA and dental caries. Concerning the association between dental caries and the hematological markers there was a very low certainty of evidence. This is due to the observational nature of included studies, risk of bias, and heterogeneity in studies. In the meta‐analyses, Aguirre‐Ipenza et al. [22] and Ji et al. [23] rated the overall evidence as very low. In contrast, Easwaran et al. [15], rated the evidence as high for association between ECC and IDA, while the certainty of evidence for the relationship between IDA and specific hematological markers was very low. To conclude, though the association appears consistent, the evidence strength remains weak.

We found that children with IDA were significantly over three times more likely to have dental caries than children without anemia (OR = 3.64). The heterogeneity was significant, but evidence of publication bias was not observed. Therefore, GRADE level was assigned as moderate for the association between IDA and dental caries. Among the included meta‐analyses of this umbrella review, Aguirre‐Ipenza et al. [22] reported the closest result to ours (OR = 3.54). Easwaran et al. [15] and Ji et al. [23] reported much higher and lower ORs of 6.07 and 2.74, respectively.

Concerning the hematological parameters, we found a statistically non‐significant difference in the levels of serum ferritin, haemoglobin and MCV between cases and controls. Except for MCV, the heterogeneity for serum ferritin and haemoglobin were significant. According to Egger's test, publication bias was significant for all the three categories. Due to this, GRADE levels were assigned as very low for the association between these three parameters and dental caries. This is interesting, as the diagnosis of IDA often depends on these laboratory parameters. Among the meta‐analyses included in this umbrella, Easwaran et al.'s [15] results had the same pattern as ours, i.e., non‐significant difference for all the three hematological parameters. In contrast, Amrollahi et al. [24] reported that all three blood parameters were significantly lower in cases of IDA. Ji et al. [23] reported significant differences for haemoglobin and MCV but not for serum ferritin. We believe the reason for this lies in a difference in the definition of IDA and the guideline for its diagnosis. If at least two of the three criteria (MCV, serum ferritin and haemoglobin) are below normal, the WHO considers IDA to be established [24]. The primary studies showed great variability in this regard. For example, Deane et al. [57] and Koppal et al. [36] used all the three parameters to define IDA [21, 36, 56, 57], Ji et al. [55] used Hb + MCV, Nayak et al. [58] used Hb + Ferritin, Jayakumar et al. [35] only used ferritin, Canchari et al. [19] only used haemoglobin and some others even have not clarified their mode of diagnosis [34, 59]. Additionally, no standardised cut‐off values and varied sample population may have also contributed to the mentioned pattern.

Only one study compared different study designs. Aguirre‐Ipenza et al. [22] reported ORs of 5.20 and 3.06 for case‐control and cross‐sectional studies respectively for the relation between IDA and dental caries. Most of the meta‐analyses did not perform an age‐based subgroup analysis, since the age of the individuals in the primary studies were mostly between 2 and 6 years with very overlapping edges. Aguirre‐Ipenza et al. [22], found significant variations based on age. They observed the association in children under 6 years and the absence of the same in children aged 6–12 years. They hypothesised that during the early years of life; iron deficiency can directly impact dental development due to being a critical phase of growth and mineralisation. Children under 6 years, in full development, depend on nutrition for oral health, and iron deficiency can compromise dental enamel, increasing susceptibility to caries. In contrast, older children may have better oral hygiene habits and access to preventive measures, reducing the impact of IDA.

Yue et al. [60], in their meta‐analysis, they investigated whether individuals with sickle cell anemia (SCA) have more dental caries than individuals with non‐SCA. They discovered that dental caries was not worse in SCD patients than in non‐SCA people. However, it is worth to note that this meta‐analysis was mainly focused on adults and for this reason it was not included in the analysis of this umbrella review [60].

Sharifi et al. [16], in their meta‐analysis, found that serum levels of ferritin were significantly different between children with and without caries, whereas ferritin and iron levels in saliva were not different. They concluded that serum blood samples, but not saliva samples, provide reliable information regarding the present levels of ferritin and iron in children with or without dental caries.

Based on the evidence from literature, there is a mutual interdependency and bidirectional association between dental caries and IDA. The summary of this association is the increased risk of one variable with the presence or increase in the odds of the other.

Several theories and biological mechanisms have been proposed to explain this association:

1. Iron deficiency often impairs salivary gland function, influencing salivary secretion and buffering capacity versus dental caries [61, 62]. Reduction in saliva production and its functions of cleaning, lubrication, and remineralization, increase the risks of dental caries. Additionally, some medications used to treat IDA, such as iron supplements, may have side effects that affect oral health and salivary function [63].
2. Iron insufficiency causes a reduction in ferric ions in the blood. Iron possesses anti‐caries properties. By joining forces with calcium and phosphate ions, iron ions can enhance the minerals dissolved in the acidic environment and suppress the function of Streptococcus mutans’ virulence factor [64, 65, 66]. Iron is essential to the crystallisation of hydroxyapatite because it promotes crystal formation and helps teeth retain their good mechanical qualities. Enamel hypoplasia may result from low iron levels [15, 67].
3. Children with dental caries are experiencing pain and difficulty in chewing. Their chewing habits may change as a result of this discomfort, and they may eat less meat and fruit, which could impact their intake of iron and eventually leading to nutritional IDA [21, 68].
4. Cytokine production brought on by inflammation in ECC can impair erythropoiesis and lower iron and haemoglobin levels. Chronic dental abscesses and pulpitis likely have an impact on children's growth by producing chronic inflammation, which in turn affects metabolic pathways where cytokines impact erythropoiesis [69]. For instance, erythropoiesis can be inhibited by interleukin, which has a variety of anti‐inflammatory properties. Because the bone marrow produces fewer erythrocytes as a result of haemoglobin inhibition, anemia may result [70]. Interleukins were reported to be higher in children with ECC [71].
5. Both IDA and ECC are complex diseases that are impacted by underlying socioeconomic determinants of health, including socioeconomic status (SES) [72, 73]. Prior research has demonstrated the negative effects of low SES on IDA and ECC [74, 75]. The validity of many of the primary studies included in this umbrella review may be jeopardised because SES was not considered in most of these research. Many youngsters may be on the verge of malnutrition due to the constraints of economic circumstances. In cases of chronic malnutrition, primary tooth eruption and replacement may be delayed, perhaps leading to a higher prevalence of ECC [74, 76]. Amrollahi et al. [24] in their meta‐analysis, only included case‐control studies to reduce the possible socioeconomic effects in different societies. They hypothesised that these investigations were done in communities with varying socioeconomic situations that may affect dental caries and IDA. Thus, including case and control groups in each community in the meta‐analysis reduces community differences.
6. Breastfeeding offers vital nutritional and immunological advantages in infancy and early childhood, but after the age of two, prolonged or excessive breastfeeding, combined with excessive milk intake, may increase the risk of dental cavities and IDA [77, 78]. Excessive milk consumption may contribute to dental caries by lowering oral pH and giving cariogenic bacteria a steady substrate when it is used as the main source of sustenance for longer than is advised. Furthermore, when iron‐rich complementary foods that are essential for fulfilling the growing child's iron needs are replaced by extended exclusive or dominating nursing, the risk of IDA may rise [78, 79].

**Figure 7:**
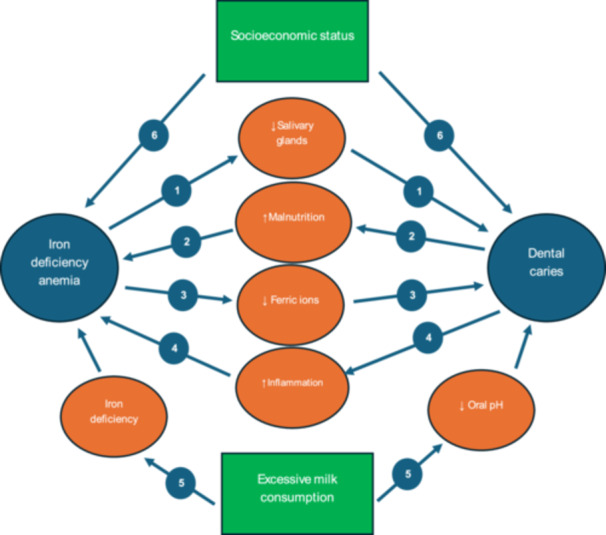
Mechanisms of association between iron deficiency anemia (IDA) and dental caries. (1) IDA impairs the function of salivary glands. A reduction in the salivary flow increases the chances of dental caries. (2) Dental caries, through pain and difficulty in chewing, decrease the intake of iron containing substances, leading to a nutritional IDA. (3) Ferric ions, which have bacteriostatic and cariostatic activities, decrease in IDA. (4) Inflammation associated with dental caries may impair normal erythropoiesis. (5) Excessive milk consumption and/or breastfeeding is a common covariate for both IDA and dental caries. It increases the odds of dental caries by providing constant substrate for oral microorganisms. It may result in nutritional IDA, since it prevents the child from taking necessary iron containing foods. (6) Low socioeconomic status is a common covariate for IDA and dental caries through nutrition/malnutrition, health awareness, access to healthcare … etc.

Figure 7 illustrates the biological mechanisms as well as risk factors and common covariates of dental caries and IDA.

Despite using the proper methods, our study had the following limitations. Cross‐sectional studies restrict the ability to demonstrate a cause‐and‐effect link. Additionally, the potential confounding influence of other known risk factors, including SES, which was not always controlled in the primary studies, limits the interpretation. The sample size, age, IDA definition and its assessment criteria, and dental caries reporting index differed throughout the studies.

### 4.1 Recommendations

Preventive dental care can lessen the severity of the condition and make treatment easier. Untreated carious processes may cause pain, which could lead to chewing difficulties and nutritional deficiencies. These factors make it possible to consider incorporating dental treatments and preventative care into anemia control initiatives.

Our findings suggest that raising awareness of the effects of IDA on dental health among medical professionals who treat children and adolescents (dentists, pediatric dentists, nutritionists and pediatricians) is essential. Specific teaching initiatives that highlight the value of holistic care that consider patients’ dental health as well as their systemic health must be put in place for these professionals.

To further understand the relationship between dental caries and IDA in children, more thorough and longitudinal study is recommended.

## 5 Conclusion

Despite the moderate certainty of the evidence, our results suggest a significant association between IDA and dental caries. Dental caries was more prevalent in children with IDA. However, the hematological parameters (haemoglobin, serum ferritin and MCV) were not statistically significant and showed high levels of heterogeneity and/or publication bias. This emphasises the need for a standardised hematological tool for the assessment of IDA. Given the inherent methodological constraints of observational designs, which are subject to biases, careful interpretation of these results is important. However, there is no doubt that this association is highly relevant to public health policies. These results could assist healthcare professionals and policymakers in identifying individuals at risk and implementing interventions to optimise outcomes in the battle against dental caries and anemia.

## Author Contributions

Balen Hamid Qadir contributed to the investigation, writing – original draft preparation, visualisation, and data curation. Mohammed Khalid Mahmood was responsible for conceptualisation, methodology, validation, writing – review and editing, formal analysis, resources, and visualisation. Yad Mariwan Mohammed Ali contributed to data curation, formal analysis, software development, and writing – original draft preparation. Tara Ali Rasheed participated in the investigation, methodology, validation, and visualisation. Zana Fuad Noori contributed to formal analysis, data curation, writing – review and editing, and validation. Handren Ameer Kurda was involved in resources, investigation, visualisation, and data curation. Mohammed Aso Abdulghafor contributed to data curation, methodology, writing – review and editing, and software. Mohammed Taib Fatih participated in the investigation, writing – original draft preparation, validation, and visualisation. Hevi Nihad Mohammed Fadhil contributed to formal analysis, data curation, writing – review and editing, and visualisation. Herve Tassery was responsible for conceptualisation, supervision, validation, and writing – review and editing. Delphine Tardivo contributed to formal analysis, methodology, supervision, and writing – original draft preparation. Romain Lan was involved in conceptualisation, project administration, supervision, and writing – review and editing.

## Conflicts of Interest

The authors declare no conflicts of interest.

## Peer Review

The peer review history for this article is available at https://www.webofscience.com/api/gateway/wos/peer-review/10.1111/jhn.70149.

## Supporting information


**Supplementary Table 1:** The search strategy of systematic search. **Supplementary Table 2:** Methodological quality assessment of the included systematic reviews and meta‐analyses using AMSTAR2. **Supplementary Table 3:** Assessment of the outcomes by GRADE.

## Data Availability

The data that supports the findings of this study are available in the Supporting Information S1: Material of this article.
