# Supplementary material for: Association Between Iron Deficiency Anemia and Dental Caries in Children: A Systematic Umbrella Meta‐Analysis
Source: J Hum Nutr Diet. 2025 Oct 27;38(6):e70149. doi: 10.1111/jhn.70149 (PMC12559779; doi:10.1111/jhn.70149)
Supplement: Supplementary file 1 — Supplementary Table 1: The search strategy of systematic search. Supplementary Table 2: Methodological quality assessment of the included systematic reviews and meta‐analyses using AMSTAR2. Supplementary Table 3: Assessment of the outcomes by GRADE. [file JHN-38-0-s001.docx]

| **Supplementary Table 1. The search strategy of systematic search** | |
| --- | --- |
| **Anemia** | “Anemia” OR “Anemia, Iron-Deficiency” OR anemia OR hemoglobin OR hematocrit OR ferritin OR “iron deficiency” OR “anemia iron deficiency” OR “low serum iron level” OR “iron-deficient anemia” OR “low iron status” |
| **Dental caries** | "Oral health" OR "dental disease" OR "dental diseases" OR "dental pathology" OR "oral pathology" OR "Dental Caries" OR “Dental Caries Susceptibility” OR “Root Caries” OR “tooth decay” OR “teeth decay” OR “cavitated caries lesion” OR “cavitated carious lesion” OR “dental caries” OR “tooth decay” |
| **Meta analysis** | “meta-analysis” OR “Meta-analysis” OR “meta analysis” OR “Meta analysis” OR "Meta-Analysis" OR "meta-analyses" OR "Meta-Analysis" OR "meta-analyze" OR "Systematic Review" OR "Systematic Reviews as Topic" OR "Meta-Analysis as Topic" |

| **Supplementary Table 2. Methodological quality assessment of the included systematic reviews and meta-analyses using AMSTAR2** | | | | | | | | | | | | | | | | | |
| --- | --- | --- | --- | --- | --- | --- | --- | --- | --- | --- | --- | --- | --- | --- | --- | --- | --- |
| **Author, year [Ref.]** | **Q1** | **Q2** | **Q3** | **Q4** | **Q5** | **Q6** | **Q7** | **Q8** | **Q9** | **Q10** | **Q11** | **Q12** | **Q13** | **Q14** | **Q15** | **Q16** | **Level of evidence** |
| Aguirre-Ipenza et al, 2024 [1] | Y | Y | Y | Y | Y | Y | N | Y | Y | N | Y | Y | Y | Y | N | Y | Critically low |
| Amrollahi et al, 2022 [2] | Y | PY | Y | Y | Y | Y | N | Y | Y | N | Y | Y | Y | Y | PY | N | Critically low |
| Easwaran et al, 2022 [3] | Y | Y | Y | Y | Y | Y | N | Y | Y | N | Y | Y | Y | Y | N | Y | Critically low |
| Ji et al., 2021 [4] | Y | Y | Y | Y | Y | Y | N | Y | Y | N | Y | Y | Y | Y | N | Y | Critically low |
| Sharifi et al., 2021 [5] | Y | Y | Y | Y | Y | Y | N | Y | Y | N | Y | Y | Y | Y | N | Y | Critically low |
| **Ref, references. PY, partially yes. Q1: Did the research questions and inclusion criteria for the review include the components of PICO?, Q2: 2. Did the report of the review contain an explicit statement that the review methods were established prior to the conduct of the review and did the report justify any significant deviations from the protocol?; Q3, Did the review authors explain their selection of the study designs for inclusion in the review?; Q4, Did the review authors use a comprehensive literature search strategy?; Q5, Did the review authors perform study selection in duplicate?; Q6, Did the review authors perform data extraction in duplicate?; Q7, Did the review authors provide a list of excluded studies and justify the exclusions?; Q8, Did the review authors describe the included studies in adequate detail?; Q9, Did the review authors use a satisfactory technique for assessing the risk of bias?; Q10, Did the review authors report on the sources of funding?; Q11, Did the review authors use appropriate methods for statistical combination of results?; Q12, Did the review authors assess the potential impact of RoB in individual studies on the results?; Q13, Did the review authors** **account for RoB in individual studies when interpreting/ discussing the results of the review?; Q14, Did the review authors provide a satisfactory explanation for, and discussion of, any heterogeneity?; Q15, Did the review authors carry out an adequate investigation of publication bias?; Q16, Did the review authors report any potential sources of conflict of interest?** | | | | | | | | | | | | | | | | | |

**Supplementary Table 3. Assessment of the outcomes by GRADE**

| **Certainty assessment** | | | | | | | **№ of patients** | | **Effect** | | **Certainty** | **Importance** |
| --- | --- | --- | --- | --- | --- | --- | --- | --- | --- | --- | --- | --- |
| **№ of studies** | **Study design** | **Risk of bias** | **Inconsistency** | **Indirectness** | **Imprecision** | **Other considerations** | **Case** | **Control** | **Relative (95% CI)** | **Absolute (95% CI)** |  |  |
| **IDA** | | | | | | | | | | | | |
| 3 | Non-randomized studies | serious | not serious | not serious | not serious | Strong association all plausible residual confounding would reduce the demonstrated effect | 3351 | 17556 | **OR 3.64** (2.45 to 5.40) | **240 more per 1,000** (from 208 more to 268 more) | ⨁⨁⨁◯  Moderate | CRITICAL |
| **Serum ferritin** | | | | | | | | | | | | |
| 4 | Non-randomized studies | serious | not serious | not serious | serious | publication bias strongly suspected all plausible residual confounding would reduce the demonstrated effect | 1862 | 1426 | - | MD **0.35 μg/L lower** (0.75 lower to 0.04 higher) | ⨁◯◯◯ Very low | CRITICAL |
| **Hemoglobin level** | | | | | | | | | | | | |
| 3 | Non-randomized studies | serious | serious | not serious | not serious | Publication bias strongly suspected strong association all plausible residual confounding would reduce the demonstrated effect | 1409 | 889 | - | MD **3.13 g/dl lower** (4.37 lower to 1.89 lower) | ⨁◯◯◯ Very low | CRITICAL |
| **MCV level** | | | | | | | | | | | | |
| 3 | Non-randomized studies | serious | not serious | not serious | not serious | All plausible residual confounding would reduce the demonstrated effect | 1545 | 813 | - | MD **0.67 femtoliters lower** (1.13 lower to 0.21 lower) | ⨁◯◯◯ Very low | CRITICAL |

**CI:** confidence interval; **MD:** mean difference; **OR:** odds ratio

References

1. Aguirre-Ipenza R, Nieto-Gutiérrez W, Contreras W, Contreras PJ, Curioso WH. Iron Deficiency Anemia and Dental caries: a systematic review and Meta-analysis. Glob Pediatr Health. 2024;11:2333794X241273130.

2. Amrollahi N, Tarrahi MJ. Iron deficiency anemia in children with and without dental caries: a systematic review and meta-analysis. Iran J Pediatr. 2022;32(4).

3. Easwaran HN, Annadurai A, Muthu M, Sharma A, Patil SS, Jayakumar P, et al. Early childhood caries and iron deficiency anaemia: a systematic review and meta-analysis. Caries Res. 2022;56(1):36–46.

4. Ji SQ, Han R, Huang PP, Wang SY, Lin H, Ma L. Iron deficiency and early childhood caries: a systematic review and meta-analysis. Chin Med J (Engl). 2021;134(23):2832–7.

5. Sharifi R, Tabarzadi MF, Choubsaz P, Sadeghi M, Tadakamadla J, Brand S, et al. Evaluation of serum and salivary iron and ferritin levels in children with dental caries: A meta-analysis and trial sequential analysis. Children. 2021;8(11):1034.
